# Synthesis and Characterization of a New Nanocomposite Film Based on Polyvinyl Alcohol Polymer and Nitro Blue Tetrazolium Dye as a Low Radiation Dosimeter in Medical Diagnostics Application

**DOI:** 10.3390/polym13111815

**Published:** 2021-05-31

**Authors:** Saleh Alashrah, Yassine El-Ghoul, Mohammed Ahmed Ali Omer

**Affiliations:** 1 Department of Physics, College of Science, Qassim University, Buraidah 51452, Saudi Arabia; ashrh@qu.edu.sa; 2 Department of Chemistry, College of Science, Qassim University, Buraidah 51452, Saudi Arabia; 3 Textile Engineering Laboratory, University of Monastir, Monastir 5019, Tunisia; 4 Department of Radiologic Technology, College of Applied Medical Sciences, Qassim University, Buraidah 51452, Saudi Arabia; ma.omer@qu.edu.sa

**Keywords:** polyvinyl alcohol, nitro blue tetrazolium, nanocomposite film, characterization, X-radiation dosimeter, medical diagnostic radiology

## Abstract

Dosimetry is a field of increasing importance in diagnostic radiology. There has been a realization among healthcare professionals that the dose of radiation received by patients via modern medical X-ray examinations could induce acute damage to the skin and eyes. The present study highlights the synthesis of polyvinyl alcohol/nitro blue tetrazolium nanocomposite films (PVA/NBT) for radiation detection depending on chromic, optical, chemical and morphologic changes. First, we synthesized the nanocomposite film-based PVA doped with NBT and the different parameters of the preparation procedure were optimized. Then The films were exposed to different low X-ray doses on the scale of mGy level (0, 2, 4, 10 and 20 mGy). The sensitivity and the performance of the made composite films were evaluated via different characterization methods. Indeed, the response curve based on UV-Vis absorptions revealed a linear increase in absorbance with increased radiation doses (R = 0.998). FTIR analysis showed a clear chemical modification in recorded spectra after irradiation. X-ray diffraction assessment revealed clear structural changes in crystallinity after ionization treatment. SEM analysis showed a clear morphological modification of PVA/NBT films after irradiation. In addition, the prepared PVA/NBT films exhibited excellent pre- and post-irradiation stability in dark and light. Finally, the quantitative colorimetry study confirmed the performance of the prepared films and the different colorimetric coordinates, the total color difference (∆E) and the color strength (K/S) showed a linear increase with increasing X-ray doses. The made nanocomposite PVA/NBT film might offer promising potential for an effective highly sensitive medical dosimeter applied for very low doses in X-ray diagnostic radiology.

## 1. Introduction

The second half of the 20th century has experienced an extraordinary industrial trend of polymer materials in all the sectors of industrial activity. Nowadays, although polymer production is continuously growing, all developed countries face new challenges regarding the development of a new generation of functional polymers—so-called smart polymers—having a vital vocation related mainly to the most important field of medicine and health [1,2,3,4,5]. Development and innovation in the fabrication of dosimeters are crucial factors for monitoring radiation exposure. Recently studied polymer nanocomposite materials for dosimeters comprise a matrix and a coloring agent [6,7,8]. Different dosimeters such as ionization chamber dosimeters, thermoluminescent dosimeters (TLDs), metal oxide semiconductor field-effect transistors (MOSFETs), films (radiographic or gafchromic), 2D array (pixel ionization chambers or diodes) and electronic portal imaging device (EPID) have been developed for radiation detection [9]. An ideal dosimeter has some basic features that make it suitable for measurements [10,11,12]. Among these basic features are reproducibility and accuracy. In fact, in dosimetry, the uncertainty related to the measurement is frequently expressed in terms of reproducibility and accuracy. High reproducibility is associated with a small standard deviation in the distribution of the results in the measurements. Reproducibility includes systematic errors in a dosimetry device, electrometer leakage and repeated measurements. The accuracy is the ability of the dosimeter to measure the delivered dose correctly and it matches the true value of the measured dose. In addition, absolute dose determination is also an important feature. An ideal dosimeter should measure the absolute dose (Gy) rather than the relative dose (%). Normalization procedure can be used to convert relative dose to absolute dose [13]. Furthermore, an ideal dosimeter response is independent of the orientation of irradiation. This requirement is ideally met if the dosimeter is tissue-equivalent and circular in shape [14]. An ideal dosimeter should also have a linear response with either the dose or the dose rate. This is important when beaten high-energy linear accelerators are used because the delivery of high-dose beats of radiation should be in short time periods. An ideal dosimeter must be independent of the field size. In addition, the response of the dosimeter should be constant when varying energies of the incident beam [15]. The smallest change in response of the dosimeter against radiation energy generally means that an effective atomic number of the dosimeter is close to the tissue [16]. Spatial resolution is also considered a desired feature. Since the dose is a point quantity, the dosimeter must be able to determine the dose from a very small volume [17]. Practically, all dosimeters have a finite size and they can provide inaccurate readings of delivered dose in high dose gradients such as the penumbral regions [18]. An ideal dosimeter must have the shortest detection time possible and the response should show the measured dose immediately after radiation, without generating any toxic hazards. Furthermore, an ideal detector must present a reasonable cost, be simple to use and be materially strong enough for clinical practice on a tedious basis without any physical limitations [14].

Evidently, all these characteristics cannot be accomplished by all dosimeters. Therefore, for the importance of the choice of the dosimeter as well as its reader, we must take into account the conditions of measurement. Some physical characteristics of an ideal dosimeter listed above have been studied for dosimeters such as gafchromic films and polymers detectors used in treatment verification or diagnostics [19,20,21].

Synthetic polymers can be synthesized with a broad variety of structures and appropriate physical and chemical properties. The increasing interest related to the design of this kind of polymers is explained by their use in a wide range of biomedical applications as diverse as tissue engineering, drug delivery, therapeutics, diagnostics, and so on [22,23,24,25,26]. Over the last decade, the methods for the synthesis of polymers, processing, and characterization have been developing rapidly, which brings both challenges and opportunities to design novel polymeric bio-materials [27,28,29,30,31].

Moreover, the hybridization of synthetic polymers by metal nanoparticles and specific selected organic dyes have been considered as a key function for the wider applications of polymer nanocomposites recently [32,33,34]. The importance of doping the polymers with selective materials can be attributed to improving the chemical and physical properties which in turn enhance the utilization of the final product in electronics, biomedical engineering, displays, physics and biosensors [35,36]. In the same realm, the doping of polymers with organic dye or compound also could induce desirable characteristics to the final products which in turn widens the applications in the field of radiation detection, measurement and biosensors.

The aim of the current study was to synthesize indigenous nanocomposite films made of PVA/NBT dye to be investigated as low radiation detection dosimeters. Preparing an efficient dosimeter for low radiation levels applied in diagnostic radiology is a veritable challenge nowadays, especially since there are no previously performed studies on using NBT for PVA film dosimetry in mGy dose levels. Firstly, we synthesized PVA/NBT film labels and the parameters of their preparation process were optimized. Then, the prepared films were irradiated with different X-ray doses (0, 2, 4, 10 and 20 mGy). The non-irradiated films and those exposed at different radiation doses were then characterized by different chemical, physical, colorimetrical and morphological techniques (UV, FTIR, densitometry, colorimetry, XRD and SEM). The comparison of the responses to the increasing doses received by irradiated PVA/NBT films via the different characterizations would be decisive in the evaluation of the performance of the nanocomposite films produced as radiation dosimeters applied in the field of X-ray diagnostic radiology.

## 2. Experimental

### 2.1. Materials

Polyvinyl alcohol (Degree of hydrolysis: 85–90% and MW: 85,000–124,000) polymer used as a matrix in the nanocomposite film and nitro blue tetrazolium chloride (C40H30 Cl2N10O6.H2O.CH4O; MW = 867.70) investigated in the development of the different film dosimeters was purchased from Sigma Aldrich (St. Louis, MO, USA). Ultrapure (Milli-pore) water (Milli-Q^®^ Direct, purified through a QPAK^®^ cartridge, Darmstadt, Germany), was used for the synthesis of the film dosimeter. All the used reagents were procured of analytical grade and were used without any further purification.

### 2.2. Characterization of Nanocomposite Films

Different techniques were performed to characterize the X-irradiated PVA/NBT film labels. The comparison between unirradiated films and those exposed to various X-radiation doses was studied to evaluate the effectiveness of the developed film dosimeters.

Virgin and X-irradiated PVA/NBT nanocomposite films were chemically characterized using FTIR spectrophotometric analysis (spectrophotometer Agilent tech with Gladi-ATR, USA). The ATR (attenuated total reflection) solid surface mode was used to carry out the different spectra. FTIR spectra were recorded from 4000 to 500 cm^−1^ with 20 scans and a resolution of 4 cm^−1^.

UV-vis absorption spectra of the different film labels were obtained using a UV–vis spectrophotometer (Shimadzu, UV-2501PC, Kyoto, Japan) in a range from 200 to 700 nm.

X-ray diffraction patterns were recorded using X-ray Diffractometer PW 1720 (Philips) with 2θ varying from 10° to 40° and a step size of 0.01°. X-ray generator was of PW 1850 was exploited as a source of radiation and was equipped with a copper tube. A nickel filter was used to generate the monochromatic radiation.

SEM analyses were performed to study the surface morphology of the PVA/NBT nanocomposite films. Micrographs were obtained using Scanning Electron Microscopy (JEOL JSM-5400 LV, JEOL Ltd., Akishima, Tokyo Japan). Analysis was realized at 5 kV of acceleration voltage and under varied magnifications from 100 to 2000×. Surface conductivity of analyzed films was improved by applying a sputter coating with a fine gold layer prior to examination.

The spectrocolorimitric study of the PVA/NBT films before and after irradiation was accomplished via a spectrocolorimeter 3NH YD5010 within the visible spectrum at 39 wavelengths with 10 nm interval from 380 nm to 780 nm. The K/S (color strength) values were determined using the Kubelka–Munk equation (Equation (1)) [37].

(1) $${\left(\frac{K}{S}\right)}_{\lambda }\;=\frac{{\left(1-{\;R}_{\lambda }\right)}^{2}}{2\times {\;R}_{\lambda }}$$

where K and S are the absorption and scattering coefficients, respectively, and R_λ_ is the spectral reflectance of the dyed fabric at λmax [38]

CIELab coordinates of colored PVA/NBT films (L*, a*, b *) and the total color difference (ΔΕ) were measured under 10 degrees standard observer and D65 standard illuminant. Where L* describes lightness, a* represents redness-greenness, b* represents yellowness-blueness [39,40].

The following Equation (2) allowed the ΔΕ according to the different color coordinates:

(2) $$\Delta E\;=\sqrt{{\left(L^{*}-{\;L}_{0}\right)}^{2}+{\left(a^{*}-{\;a}_{0}\right)}^{2}+{\left(b^{*}-{\;b}_{0}\right)}^{2}}$$

where (L*, a*, b*) and (L_0_, a_0_, b_0_) are the color coordinates for the unirradiated and irradiated PVA/NBT films respectively. The average limit of the total color difference between two color points is the value above which the human eye cannot differentiate. Many limits depending mainly on color saturation have already been published in the literature [41,42].

### 2.3. Preparation of PVA/NBT Nanocomposite Films

PVA/NBT film samples were prepared via a simple cast method. A bulk Polyvinyl alcohol solution was prepared by dissolving 5 g of PVA in 100 mL of Milli-pure water. The solution was then kept in continuous stirring for 2 h at 80 °C. The obtaining homogenous solution was then cooled to ambient temperature. A prepared solution of NBT (0.04 g) dissolved in 10 mL of ethanol was added to the PVA solution. The mixing solution was stirred at ambient temperature for 1 h. Film labels were casted by pouring 20 mL of the prepared solution onto each glass Petri plate using a graduated syringe. Films were then set to dry in darkness at room temperature for 72 h. Uniformly dried transparent films were peeled out of the Petri dishes. The average thickness of the prepared films was then measured using a thickness gauge and was around 100 μm. Films were finally cut into small square-shaped pieces with 2 cm on the side and stored in sealed black envelopes in dark to avoid light degradation and to achieve a thermal equilibrium before investigations.

Films were finally exposed to different radiation doses using a digital X-ray fluoroscopic machine model (GE, healthcare, model Al01CII, 2011-German, Chicago, IL, USA). This machine was adjusted depending on the exposure technique factors (kVp = 60, mAs = 16 and at 100 cm) to produce exposure doses of x, y, z mGy. The system was calibrated and ascertained by an ionization chamber to produce the relevant doses.

## 3. Results and Discussion

### 3.1. Post-Irradiation Stability

**Figure 1:**
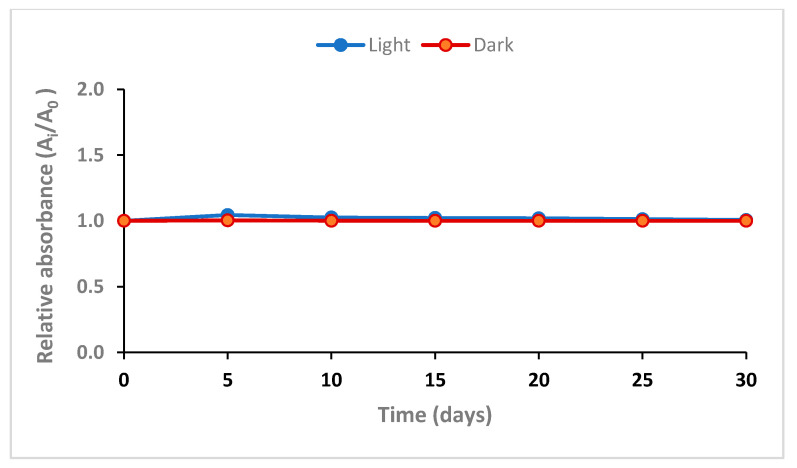
Post-irradiation stability of PVANBT films stored in dark and light at room temperature, λmax = 430 nm.

PVA/NBT-made films exposed to a dose of 20 mGy were stored in dark and light at room temperature in the laboratory. Films were stored for 30 days and the absorbance was measured during varied intervals of storage time at 352 nm. Figure 1 shows the variation of the absorbance capacity according to the time of storage after X-irradiation of the PVA/NBT nanocomposite films.

Results in this figure revealed the good stability of the films for the different periods of storage. The excellent stability was confirmed for both films stored in dark and in light, until the end of the storage period of 30 days. The studied stability upon storage period and conditions was excellent and efficient compared to other dosimeter using PVA and NBT in form of a solution or gel [43,44] which, showed weak stability especially when stored under light. This could be explained by the casting in form of the film allowing more crystalline properties. 

### 3.2. Direct Percepted Color Effect after Irradiation

**Figure 2:**
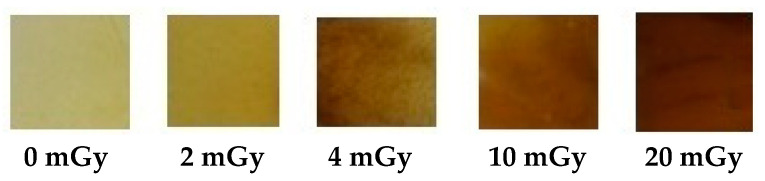
Visual color change of PVA/NBT films before and after exposure to varying doses of X-radiations.

The exposure of film nanocomposite samples of PVA dopped NBT to X-irradiation from 0 to 20 mGy resulted in a clear change in color even with low doses (Figure 2). The change in color, from transparent colorless for PVA film to clear yellow for unirradiated PVA/NBT and to yellowish-brown gradually after X-irradiation, confirmed the high sensitivity of the made nanocomposite films to low radiation doses (at the mGy scale). The formation of hydrazine due to the reduction of the formazan may be the consequence of the change in color of the films [45,46]. The further studies presented in the following will show the effect of X-irradiation on other parameters.

### 3.3. UV/Visible Absorption Spectra

**Figure 3:**
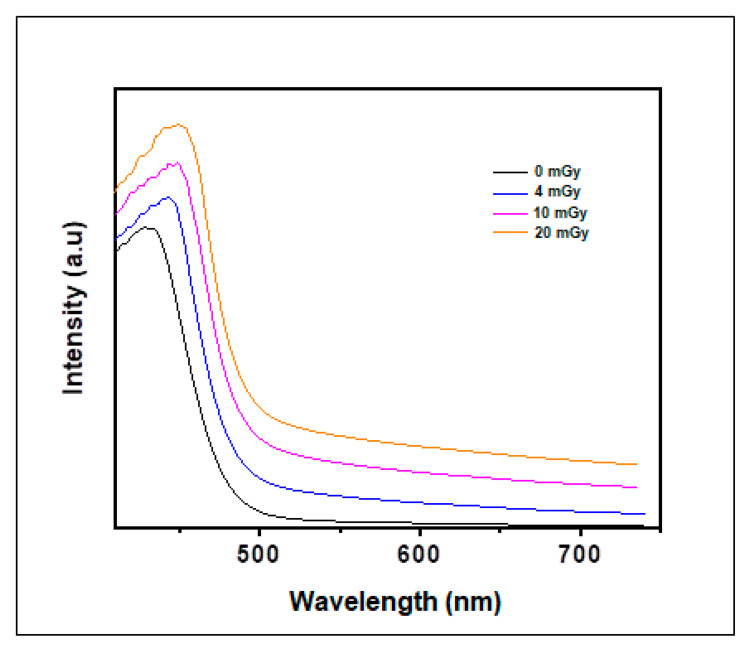
Absorbance spectra of PVA/NBT nanocomposite films before and after X-irradiation at varying doses.

Figure 3 presents the UV-vis absorption spectra of both PVA control film and those irradiated with the different X-ray doses. In the case of the PVA film which is transparent and colorless, there is no absorption peak in the visible region. The film is seen to become yellowish-brown gradually after X-irradiation. Results in Figure 3 show one sharp band with maximum absorption at 430 nm. The absorption capacity of these bands increased according to the augmentation of the applied doses. The gradual increase noted in the broadening peak with the absorbed dose is related to the reduction of NBT2+ to mono-formazan (MF+) then to a stable hydrophobic di-formazan (DF) structure under the effect of the applied irradiation [47,48]. The region of maximum absorption is violet/blue in color which is ascribed to yellow/orange as a complementary color. Therefore, the visual observation in color change presented previously is confirmed via the UV-spectroscopic analysis by observation of the absorption peak at 430 nm. Furthermore, the absorption maximum was shifted gradually with the increasing dose from 430 to 450 nm (in the case of 20 mGy dose). This was probably due to the more pronounced reduction of formazan for the NBT and the appearance of the di-formazan product.

### 3.4. Response Curve

**Figure 4:**
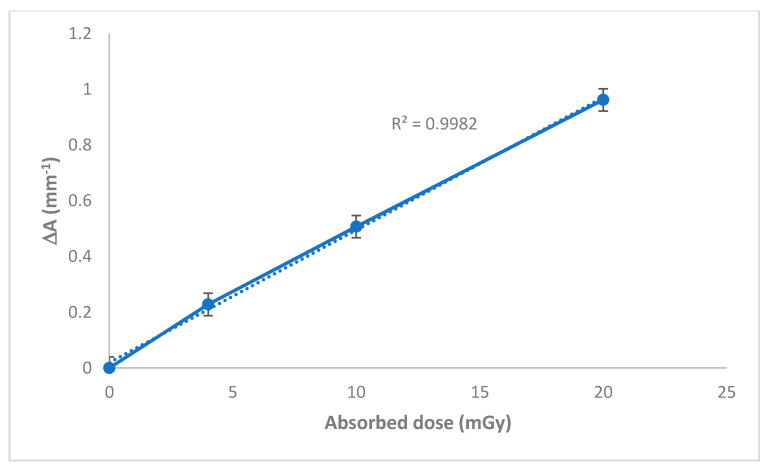
Dose–response curve based on UV-Vis absorption capacities at λmax for PVA/NBT films exposed at various X-ray doses.

Figure 4 shows the response curve of the nanocomposite PVA/NBT films exposed to different X-ray doses. The response curves were established in terms of change in absorbance per unit thickness, ΔA.mm^−1^ versus the absorbed dose, where ΔA = A0 − Ai, A0 and Ai are values of absorbance for the unirradiated and irradiated film respectively at 430 nm. The resulting curve shows a linear increase in absorbance as a function of the increase in X-ray doses with R = 0.998 as the coefficient of linear regression. This result is very important in the application of the PVA/NBT synthesized films as dosimeters of high sensitivity, in particular at very low radiation doses.

### 3.5. FTIR Analysis

The reduction of colorless to slightly yellowish tetrazolium salts using reducing agents as ionizing radiations produces a deeply colored mixture of formazan structures. Formazans exist in multiple conformers as tautomers and stereoisomers make the investigation analysis of their physical and chemical properties more complicated [49].

**Figure 5:**
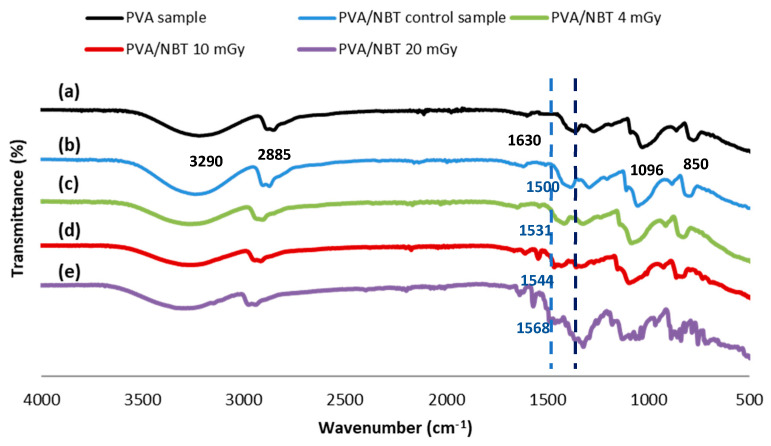
FTIR spectra of PVA film (**a**), unirradiated PVA/NBT film (**b**) and the X-irradiated PVA/NBT films receiving different doses; 4 mGy (**c**), 10 mGy (**d**) and 20 mGy (**e**).

As explained above, the effect of the applied irradiation could be assigned by the transformation of the formazan structure to the other reduced structures. FTIR spectroscopy characteristics for formazans are the C=N, N–H and N=N absorption bands. Possible open structures after reduction show higher frequencies for these bands, due to the loss of resonance stabilization of the six-membered chelate ring. Figure 5 shows the different FTIR spectra of PVA, unirradiated PAV/NBT and irradiated PVA/NBT films with various X-ray doses. A characteristic infrared spectrum for PVA casted film is presented in Figure 5.

The different typical absorption peaks and their referring chemical groups are summarized in Table 1.

A new peak appeared after the addition of NBT in the PVA film, centered at 1505 cm^−1^ referring to the C=N stretching band which was ascribed to the formazan chelates [55,56]. This absorption characteristic peak revealed a gradual shifting according to the increased applied doses. In addition, the intensity of the peak increased with the X-ray doses. Another characteristic peak appeared at 1397 cm^−1^ referring to the N=N stretch vibration for the reduced formazan structure [57]. This peak became more intense and shifted gradually with the increase of the X-radiation doses. Therefore, the FTIR performed characterization revealed well the stability of the PVA structure upon low X-ray doses while no chemical modification in the PVA characteristic groups (no scission in the main chain or removing of OH groups were detected). However, the effect of applied radiation appeared clearly on the nanocomposite PVA/NBT inducing some chemical transformations in the NBT structure.

### 3.6. X-ray Diffraction Study

**Figure 6:**
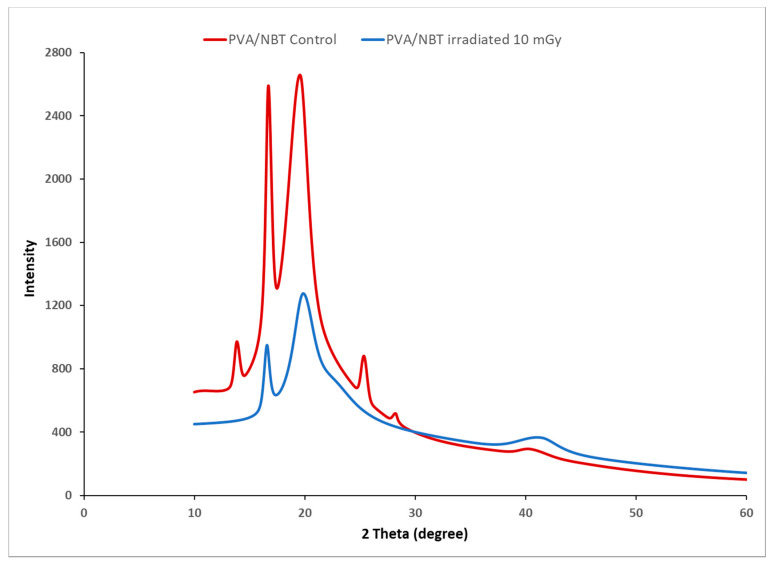
X-ray diffraction patterns of control and irradiated PVA/NBT films.

The X-irradiation effect could be confirmed by the detection of induced changes in the crystallinity of PVA/NBT composite films. X-ray diffraction analysis was performed to evaluate the changes in crystallinity illustrated in the XRD patterns before and after x-radiation exposure of the made films to different doses. Figure 6 shows X-ray diffractograms for control and X-irradiated PVA/NBT nanocomposite films at various doses. Control PVA/NBT film exhibited a typical peak centered at 2θ = 19°, which is ascribed to diffraction from a mixture of planes (101) and ($10\bar{1}$) [58]. XRD patterns showed a significant decrease of the peak intensity at 2θ = 19° from control to X-irradiated films with 20 mGy. Furthermore, the results revealed the presence of two characteristic peaks on the diffractogram of the control film at 2θ = 14 and 17°. These peaks were assigned to the diffraction from (100) and $(00\bar{1}$) planes of PVA. We remarked on the disappearance of the two last-mentioned peaks after the X-irradiation of the PVA/NBT film. The peak at 2θ = 41.06° also corresponds to the PVA crystalline phase [59]. The different recorded changes in the intensity of the various peaks reveal that a structural rearrangement occurred after irradiation.

### 3.7. SEM Morphological Analysis

**Figure 7:**
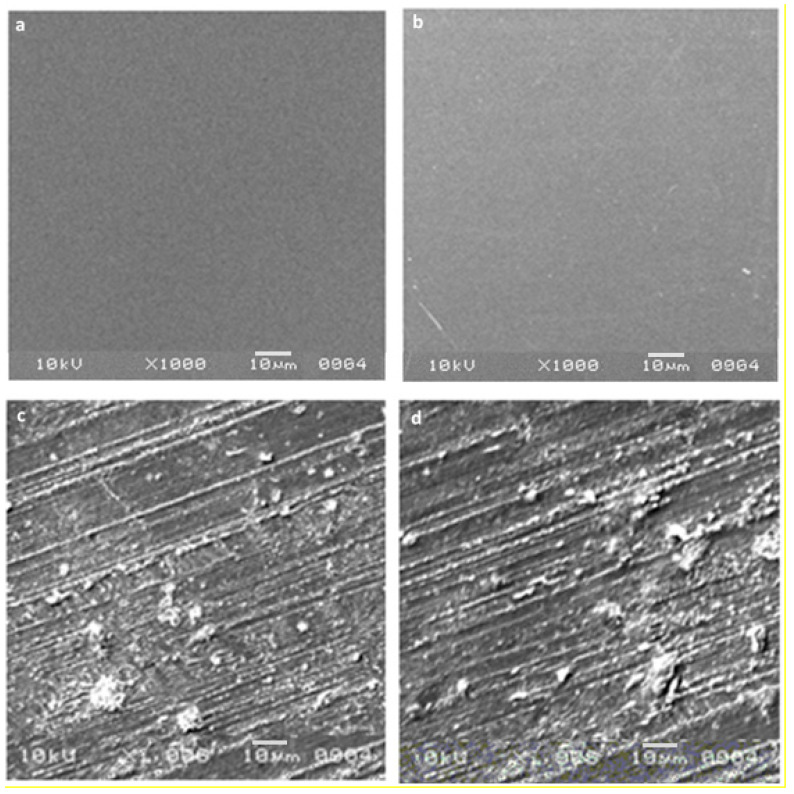
Scanning electron microscopy (SEM) morphologies of pure PVA film (**a**), unirradiated PVA/NBT (**b**) and irradiated PVA/NBT films with 10 mGy (**c**) and 20 mGy (**d**).

Morphological SEM analysis of control and irradiated PVA/NBT films was performed to evaluate the effect of the low X-ray doses on the surface morphology of the exposed samples. Figure 7 revealed for the PVA/NBT unirradiated film a smooth and homogeneous appearance with good structural integrity and crack-free states. The same observations were noted with the PVA films [60]. This obtained surface morphology showed the high compatibility of the two mixed compounds (PVA and NBT) and a compact structure lacking phase separation. After X-irradiation of the PVA/NBT films, we noted some fluke-like structures with the appearance of globules and many surface stripes. The various modifications and changes on the surface morphology of treated films became more intense with the increase of the X-ray applied dose. The clear change in surface morphology after irradiation revealed the high sensitivity of the PVA/NBT prepared films and their effective application as dosimeters for low radiation, finding their interest in various medical diagnostic applications.

### 3.8. Colorimetric Study

Change in color shade or intensity is a very important practical property for efficient dosimeters. A colorimetry study was performed to evaluate and quantify the color deviation of the PVA/NBT films after their exposure to different low radiation doses. The mean deviation of the CIELab coordinates L*, a*, b*, the ΔE (total color difference) and the color strength (K/S) values for the unirradiated and the irradiated PVA/NBT-made films are summarized in Table 2.

**Figure 8:**
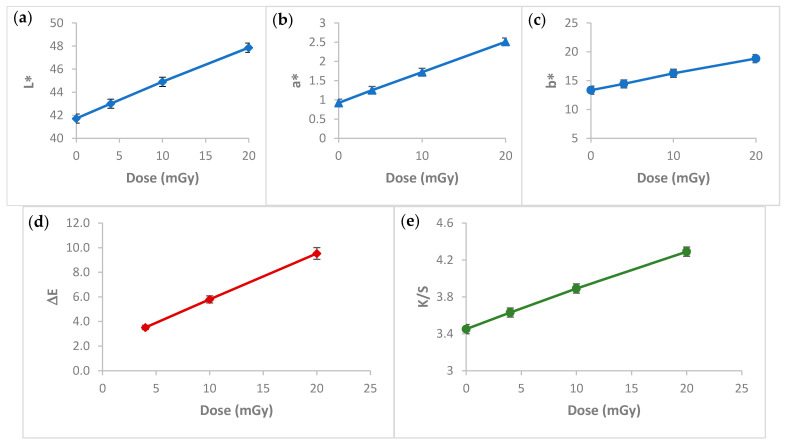
Hunter’s color parameters: (**a**) lightness L*, (**b**) redness-greenness axis a*, (**c**) yellowness-blueness axis b*, (**d**) total color difference ΔE and (**e**) color strength K/S values for PVA/NBT films irradiated from 4 to 20 mGy.

Figure 8a–c shows the variation of the CIELab coordinates as a function of the radiation dose applied to the PVA/NBT films. L* decreased with increasing doses, this indicates that the color of the films has become darker. However, a*, b* values increased with doses so the films became more reddish and more bluish. We remarked for all these parameters a linear variation with a high correlation of R = 0.99. The linear dependence of the different color coordinates according to the increased applied radiation dose allows each color’s coordinate index a*, b* and L* to be investigated independently as a dosimetry index in the low dose range from 0 to 20 mGy. In addition, Figure 8d revealed the increase of the total color difference ΔE values with the X-rays applied doses. We remarked also a linear change of the calculated ΔE according to increased radiation doses. The same was observed for the color intensity or color strength parameter K/S, which increased linearly with increasing X-ray doses (Figure 8e). The colorimetry study revealed the importance of the made nanocomposite PVA/NBT film to be applied as an efficient irradiation indicator and its potential application as a new dosimetric system in the field of X-ray diagnostic radiology.

## 4. Conclusions

Assessment of the X-ray dose to the patient is an essential aspect of the optimization process in X-ray diagnostic radiology. Therefore, finding an effective dosimetric system for low radiations in X-ray diagnostic radiology is a great challenge nowadays. The prepared PVA/NBT nanocomposite film showed high sensitivity upon various chemical, physical, morphological and colorimetrical characterizations after its exposure to increased low doses of X-radiations. Indeed, the results showed a gradual increase in the absorption capacity of the made PVA/NBT films with increased applied doses. This was related to the reduction of NBT+2 to mono-formazan configuration then to a more stable hydrophobic di-formazan structure under the effect of the applied irradiation. The resulting response curve based on UV-Vis absorptions revealed a linear increase in absorbance with the augmentation of the X-ray low doses. The FTIR chemical characterization confirmed the presence of the effect of applied radiations on the nanocomposite PVA/NBT via the apparition of new bands ascribed to the chemical transformations in the NBT structure. X-ray diffraction analysis revealed clear changes in crystallinity illustrated in the XRD patterns after irradiation of the made films. The different recorded changes in the intensity of the various peaks are due to a structural rearrangement that occurred after irradiation. SEM analysis showed a clear morphological modification of PVA/NBT films after irradiation showing their high sensitivity upon very low X-radiations. In addition, the prepared PVA/NBT films exhibited excellent pre- and post-irradiation stability in dark and light from 5 to 30 days after radiation exposure. More important results were established with the colorimetry study since a clear visual change in color appears after irradiation treatment. This color change was evaluated quantitively by determining the different colorimetry coordinates, the total color difference and the color strength. All these parameters were showed to be increased linearly with increasing X-ray doses. The different characterization analysis confirmed the high sensitivity of the prepared films upon very low X-ray doses (in the scale of mGy). Furthermore, they revealed the practical efficiency of this nanocomposite PVA/NBT film to be applied as an effective irradiation indicator and its potential application as a new dosimetric system for routine diagnostic medical process control. 

## Figures and Tables

**Table 1:** FTIR attribution bands of PVA control film.

FTIR Bands (cm^−1^)	Attribution	Assignment	Reference
850	C-H stretching	PVA backbone	[50]
916	CH_2_ rocking	PVA backbone	[50,51]
1096	C-O stretch vibration	PVA acetyl group	[50]
1141	C-O-C stretch	Polysaccharide’s pyranose	[50,51,52]
1630	C-OH bending vibration	PVA hydroxyl group	[53]
2850–2893	C-H stretch vibration	PVA backbone	[54]
3290	OH stretch vibration	PVA hydroxyl group	[54]

**Table 2:** Mean deviation of the CIELab coordinates L*, a*, b*, the ΔE and K/S values for unirradiated and X-irradiated PVA/NBT films from 4 to 20 mGy.

Sample Dose	L*	a*	b*	ΔE	K/S
0	41.71	0.92	13.34		3.4
4	43	1.25	14.44	3.508	3.63
10	44.9	1.72	16.27	5.793	3.89
20	47.86	2.51	18.83	9.528	4.29
